# Utility of a modified vascular corrosion casting technique in the diagnosis of fetal total anomalous pulmonary venous connection

**DOI:** 10.1038/s41598-021-90681-x

**Published:** 2021-05-28

**Authors:** Jiaqi Zhang, Wei Feng, Qiaoyue He, Hongzhi Guo, He Zeng, Ziyi Si, Ya Liu, Yu Wang

**Affiliations:** 1 Department of Ultrasound, Xiangyang No. 1 People’s Hospital, Hubei University of Medicine, 15 Jiefang Avenue, Xiangyang, 441000 Hubei China; 2 Xiangyang Key laboratory of Maternal–Fetal Medicine in Fetal Heart Diseases, Hubei, China

**Keywords:** Diseases, Cardiology, Cardiovascular biology, Cardiovascular diseases, Heart development

## Abstract

Total anomalous pulmonary venous connection (TAPVC) is a rare congenital cardiac malformation, and prenatal detection of TAPVC malformation remains a challenging. TAPVC can be easily missed or misdiagnosed in prenatal examinations. This study was aimed to use the modified vascular corrosion casting technique to prepare fetal cardiovascular casts with TAPVC and investigate the utility of cardiovascular casting for the demonstration of fetal TAPVC. The retrospective study enrolled twenty fetuses (22 to 29 + 4 gestational weeks) with TAPVC diagnosed by prenatal echocardiography and casting technique from May 2015 to May 2020. Pre- and postnatal medical records, including results obtained by prenatal ultrasound, postpartum computed tomography angiography, as well as anatomic and cardiovascular casting findings were carefully reviewed and analyzed. In twenty cases, 80% (16/20) had intra- or extracardiac malformations. The TAPVC types were supracardiac (n = 8), cardiac (n = 6), infracardiac (n = 4), and mixed (n = 2). The diagnosis of 1 case each of supracardiac and cardiac TAPVC was modified to partial anomalous pulmonary venous connection; additionally, 4 malformations were missed and 2 were misdiagnosed, including an anomalous left brachiocephalic vein in supracardiac TAPVC, abnormal inflow of the hepatic vein and a double inferior vena cava in infracardiac TAPVC; and bilateral ductus arteriosus in infracardiac TAPVC; a tetralogy of Fallot in cardiac TAPVC that was corrected to right ventricular double outlet; and an absence of ductus arteriosus that was misdiagnosed as slim ductus arteriosus. Comparing with ultrasound, casting technique has its own superiority in exhibiting TAPVC abnormalities, especially in certain types such as course, origin and absence abnormalities of ductus. Postpartum cardiovascular casts can accurately depict the branch structure of the heart’s larger vessels, and may be used as a clinical assessment and teaching method in complex cardiac malformations.

## Introduction

Total anomalous pulmonary venous connection (TAPVC) is a rare condition that accounts for 0.7%–1.5% of cases of congenital heart disease^1^, with a reported incidence of approximately 7–9 per 100,000 in live births^2,3^. The main feature of this condition is that all 4 pulmonary veins (PVs) fail to form a direct connection with the left atrium, instead draining into the right side of the heart via different routes of systemic venous return^4^. Examination of the fetal venous system has become an increasingly important aspect of prenatal monitoring, because isolated venous abnormalities are not only potentially fatal, but may also reflect more extensive heart malformations or genetic syndromes^5,6^. TAPVC is the only condition among cyanotic heart diseases that involves venous malformation, and it is easily misdiagnosed^7^. Early diagnosis of TAPVC—especially prenatally—is critical because after birth, cyanosis often occurs through the mixing of blood in the systemic and pulmonary circulations, which may be accompanied by PV reflux obstruction^8^ and lead to a poor outcome without timely intervention. Early diagnosis of abnormal venous drainage is also an important consideration for surgery^9–12^. Recognizing TAPVC in utero remains clinically challenging for obstetricians; in a retrospective analysis of birth records from 1998 to 2004, only 1.9% of TAPVC cases (8 in 424) were prenatally diagnosed^13^.

Because of its noninvasiveness and convenience and real-time monitoring capabilities, prenatal ultrasound is becoming the preferred method for diagnosing TAPVC, especially with the development of new scanning technologies such as color flow Doppler imaging and spatiotemporal image correlation, which has improved the diagnosis rate. However, given the lack of awareness of this rare malformation and the fact that it is often accompanied by complex cardiac and intracardiac malformations, TAPVC can be easily overlooked or misdiagnosed^14^.

In the present study, we retrospectively analyzed the clinical data of 20 cases of fetal TAPVC diagnosed by prenatal ultrasound, and compared the results of antenatal echocardiography with postpartum echocardiography, postpartum computed tomography angiography (CTA), and anatomic findings to establish the characteristics of different types of TAPVC and their venous return. In order to clearly display the 3D structure of fetal vessels, modified cardiovascular casts of some specimens were also used for postpartum diagnosis.

## Materials and methods

### Study participants

The study was approved by the ethics committee of Xiangyang No. 1 People’s Hospital Affiliated with Hubei University of Medicine. All pregnant women enrolled in our study provided written, informed consent. We retrospectively analyzed 20 cases of TAPVC diagnosed by ultrasound before delivery between May 2015 and May 2020. The gestational age ranged from 22 + 4 to 29 + 6 weeks (mean, 25 + 3 weeks), and maternal age was between 19 and 36 years (mean, 27.6 years).

### Prenatal ultrasound

Prenatal ultrasound was performed according to guidelines of the International Society of Ultrasound in Obstetrics and Gynecology and American Society of Echocardiography using a Voluson E8 or 730 ultrasound system (GE Medical Systems, Zipf, Austria) equipped with RM6C, C4-8, and C4-8D transducers (4–8 MHz). The number, location, and specific PV drainage routes were visualized in a short axis section of the heart.

### Postnatal diagnosis

Postpartum diagnosis was based on ultrasound, CTA, surgery, anatomic examination, and cardiovascular casts. For pregnant women who chose natural delivery, postpartum diagnosis was made according to the results of the operation or postpartum ultrasound. Some pregnant women chose to have labor induced because of complex intra- or extracardiac malformations in the fetus, and donated the specimens to our maternal and fetal medical center. In these cases, the postpartum diagnosis was based on CTA, anatomy, and cardiovascular casts. Postpartum ultrasound was performed using a GE Vivid 7 ultrasound system (GE Medical Systems, Zipf, Austria) with a probe frequency of 2.5–12 MHz. Reflux into the left atrium and cardiac malformations were visualized by analyzing Cardiac segments and aortic cross sections of the superior sternal fossa.

### Modified cardiovascular casting

For cardiovascular casting, the specific steps are as follow: (1) Fill in the fetal basic information record form and documentary; (2) The fetal weight, body length, head circumference, chest circumference, abdominal circumference, eye distance, upper limb length and lower limb length were measured and recorded; (3) Take photos before casting; (4) Incision of abdominal wall, separation of umbilical vein, umbilical vein intubation; (5) The left umbilical artery was cut off; (6) Heparin (5–10 ml) and acetone (20–50 ml) were injected through umbilical vein catheterization to wash blood and blood clots in cardiovascular system. The injection amount of acetone was adjusted according to the situation of blood washing until no blood flowed out. The mixture of self-coagulating denture powder and denture water was perfused slowly with pressure of 30 ~ 60 ml, and the injection time was controlled at about 30 min. Finally, the mixture of self-setting denture powder and denture water was infused with a pressure syringe for about 10 h; (7) After 24 h, the fetal specimens perfused with the mixed reagent of self-setting denture powder and denture water were immersed in 30% hydrochloric acid solution; (8) After about 2 weeks, the perfused fetal specimens were taken out from hydrochloric acid solution, and the corroded soft tissues were washed carefully with running water. The soft tissues that were not washed clean were washed carefully with syringe until there was no soft tissue on the surface of cardiovascular cast; (9) The fetal cardiovascular cast specimens were registered and photographed. The ratio of materials used for perfusion was as follows: (1) Denture powder: Denture water: dibutyl phthalate: barium sulfate = 20:40:6:6; (2) The total amount of perfusion reagent used for perfusion: 30 ~ 60 ml; (3) Speed of filling: 0.5 ml/s; (4) The temperature of perfusion environment: room temperature 20–25 ℃; (5) Humidity of perfusion environment: 50% ~ 70%; (6) Concentration of hydrochloric acid in acid tank: 30%.

### Ethical approval

Informed consent was drawn from all patients. All methods were carried out in accordance with relevant guidelines and regulations. The study was approved by the ethics committee of Xiangyang No. 1 People’s Hospital Affiliated with Hubei University of Medicine with reference number XYFH20150124, and date of approval January 24, 2015.

## Results

### General and clinical characteristics of cases

A total of 20 fetuses with a prenatal diagnosis of TAPVC were included in the analysis. The median age of the pregnant women was 27.6 years (range, 19–36 years), and the median gestational age at diagnosis was 25 weeks (range, 22–29 weeks). Of the 20 cases, 18 were confirmed as TAPVC by postpartum diagnosis, and the other 2 (cases 4 and 15) were modified to partial anomalous pulmonary venous connection. The different types of TAPVC were supracardiac, 44% (8/18); cardiac, 22% (4/18); infracardiac, 22% (4/18); and mixed, 11% (2/18). Intra- or extracardiac malformations were observed in 78% (14/18) of cases, and 22% (4/18) were confirmed as isolated TAPVC. Among TAPVC fetuses with intra- or extracardiac malformations, 78% (11/14) showed atrial isomerism, 43% (6/14) had pulmonary artery stenosis, 28% (4/14) had a single ventricle, 21% (3/14) had an endocardial cushion defect, 14% (2/14) had a single atrium, and 21% (3/14) had a right aortic arch. Four malformations were misdiagnosed, including an anomalous left brachiocephalic vein in supracardiac TAPVC (case 3), abnormal inflow of the hepatic vein and a double inferior vena cava in infracardiac TAPVC (case 9); and bilateral ductus arteriosus in infracardiac TAPVC (case 14). Two malformations were misdiagnosed, including a tetralogy of Fallot in cardiac TAPVC that was corrected to right ventricular double outlet (case 11); and an absence of ductus arteriosus that was misdiagnosed as slim ductus arteriosus (case 13) (Table 1).

**Table 1:** Prenatal diagnosis, postnatal characteristics, and outcome of 20 cases with prenatally diagnosed TAPVC.

Case	Maternal age (year)	Gestational age (week)	Prenatal diagnosis	Mianly associated malformations	Postnatal diagnosis	Diagnosis at postnatal echocardiography/operation/autopsy/casting	Outcome
1	36	25	Supracardiac TAPVC	RAI, SA, SV, DOSV, PAS, RAA with mirror branches, RDA, slim DA, dextrocardia	Confirmed	Supracardiac TAPVC	TOP
2	26	23	Cardiac TAPVC	AIS, ECD, SV, PTA, PLSVC	Confirmed	Cardiac TAPVC	A&W
3	32	29	Supracardiac TAPVC	AIS, DcORV, PAS, PLSVC, anomalous LCV	Modified	Supracardiac TAPVC;anomalous LCV was misdiagnosed	A&W
4	28	24	Cardiac TAPVC	RAI, ECD, SV, IAA	Modified	PAPVC with 1 PV -rsvc-ra to RA, 3 PV to LA	A&W
5	32	25	Cardiac TAPVC	RAI, ECD, PAS, RAA with mirror branches, PE	Confirmed	Cardiac TAPVC	A&W
6	21	26	Supracardiac TAPVC	AIS, PS, functional SV,DOSV	Confirmed	Supracardiac TAPVC	TOP
7	26	25	Supracardiac TAPVC	RAI, SV, PTA	Confirmed	Supracardiac TAPVC	TOP
8	28	23	Infracardiac TAPVC	RAI, diaphragmatic hernia, SA, severe PS, pulmonary atresia, abnormal inflow of HV	Confirmed	Infracardiac TAPVC	TOP
9	23	23	Infracardiac TAPVC	RAI, VSD, abnormal inflow of HV, double ICV	Modified	Infracardiac TAPVC; abnormal inflow of HV and double IVC were missed	A&W
10	29	22	Cardiac TAPVC	No	Confirmed	Cardiac TAPVC	A&W
11	25	24	Cardiac TAPVC	RAI, ECD, PS,DORV	Modified	Cardiac TAPVC; DORV was misdiagnosed as TOF	A&W
12	21	27	Infracardiac TAPVC	No	Confirmed	Infracardiac TAPVC	A&W
13	32	26	Mixed TAPVC	Absence of DA, PAS	Confirmed	Mixed TAPVC, absence of DA was misdiagnosed as slim DA	A&W
14	34	23	Infracardiac TAPVC	LAI, ASD, bilateral DA	Modified	Infracardiac TAPVC, bilateral DA was missed	TOP
15	36	24	Supracardiac TAPVC	RAI, ECD, functional SV, DOSV, PAS, DSVC	Modified	PAPVC with 1 PV draining to RA, 3 PV to LA	TOP
16	28	29	Supracardiac TAPVC	No	Confirmed	Supracardiac TAPVC	A&W
17	22	26	Supracardiac TAPVC	AIS, levoversion, ASD, functional SV, DOSV, DISV	Confirmed	Supracardiac TAPVC	TOP
18	27	25	Supracardiac TAPVC	SV, dextrocardia, atresia of right AV, RAA	Confirmed	Supracardiac TAPVC	TOP
19	19	27	Mixed TAPVC	Aortic dysplasia, PLSVC	Confirmed	Mixed TAPVC	A&W
20	28	24	Supracardiac TAPVC	No	Confirmed	Supracardiac TAPVC	A&W

*RAI* right atrial isomerism syndrome, *SA* single atrium, *SV* single ventricle, *DOSV* double-outlet single ventricle, *PAS* pulmonary artery stenosis, *RAA* right aortic arch, *RDA* right descending aorta, *TOP* termination of pregnancy, *HV* hepatic vein, *AIS* atrial isomerism syndrome, *ECD* endocardial cushion defect, *PTA* persistent truncus arteriosus, *PLSVC* persistent left superior vena cava, *DORV*, double outlet right ventricle, *LCV* left cephalopobrachial vein, *IAA* interrupted aortic arch, *PE* pericardial effusion, *HV* hepatic vein, *VSD* ventricular septal defect, *IVC* Inferior vena cava, *DSVC* double superior vena cave, *DISV* double inlet single ventricle, *AV* atrioventricular valve, *A&W* alive when writing.

### Postpartum modified cardiovascular casting diagnosis

**Figure 1:**
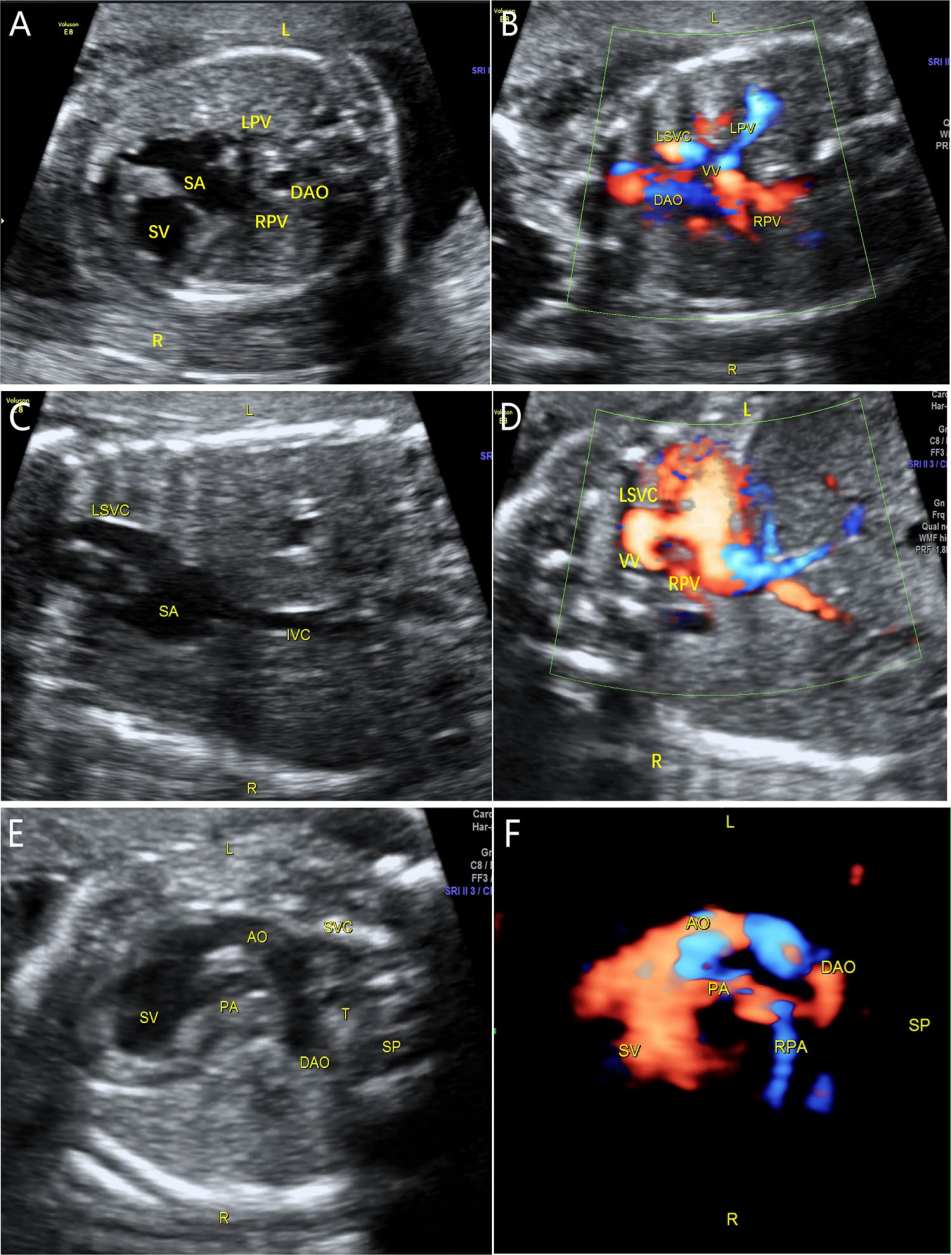
Prenatal ultrasound diagnosis of fetal supracardiac TAPVC with complex malformations. (**A**) The common PV trunk was visible in the cross section of the atrium, and posterior left atrium index was increased. (**B**) Vertical venous return to the left superior vena cava. (**C**, **D**) Superior and inferior vena cava return to a single atrium. (**E**, **F**) Two- and 3-dimensional flow imaging revealed both the aorta and pulmonary artery originating from a single ventricle with pulmonary stenosis. *DAO* descending aorta, *L* left, *LPA* left pulmonary artery, *R* right, *RPA* right pulmonary artery, *SA* single atrium, *SV* single ventricle.

**Figure 2:**
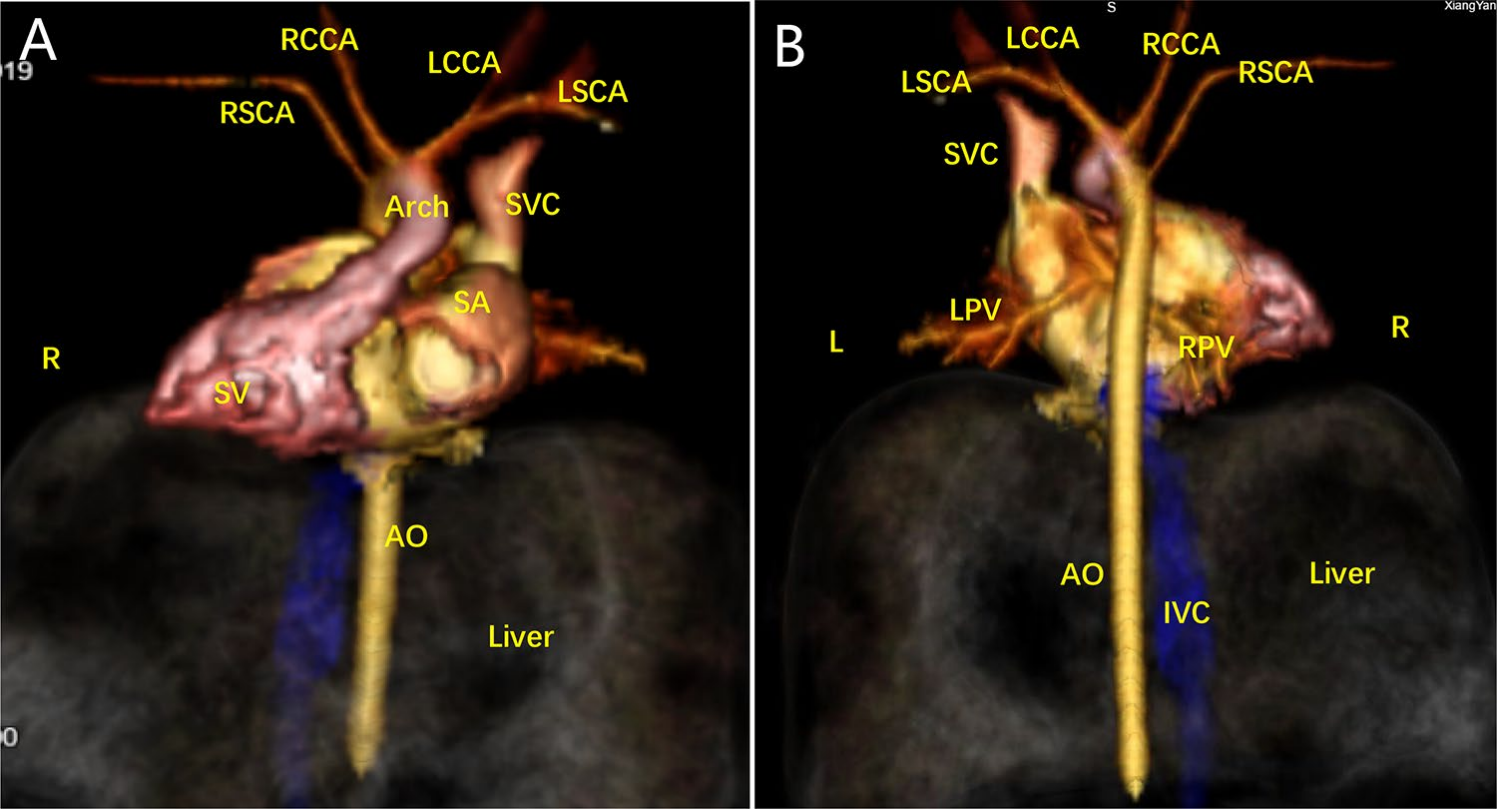
Postpartum CTA findings. (**A**–**F**) CTA revealed dextrocardia, a single ventricle, isolated left superior vena cava, right aortic arch with mirror branches, and left and right PVs returning to the left superior vena cava via vertical veins. *AO* ascending aorta, *IVC* inferior vena cava, *LCCA* left common carotid artery, *LSCA* left subclavian artery, *RCCA* right common carotid artery, *RSCA* right subclavian artery, *SA* single atrium, *SV* single ventricle.

**Figure 3:**
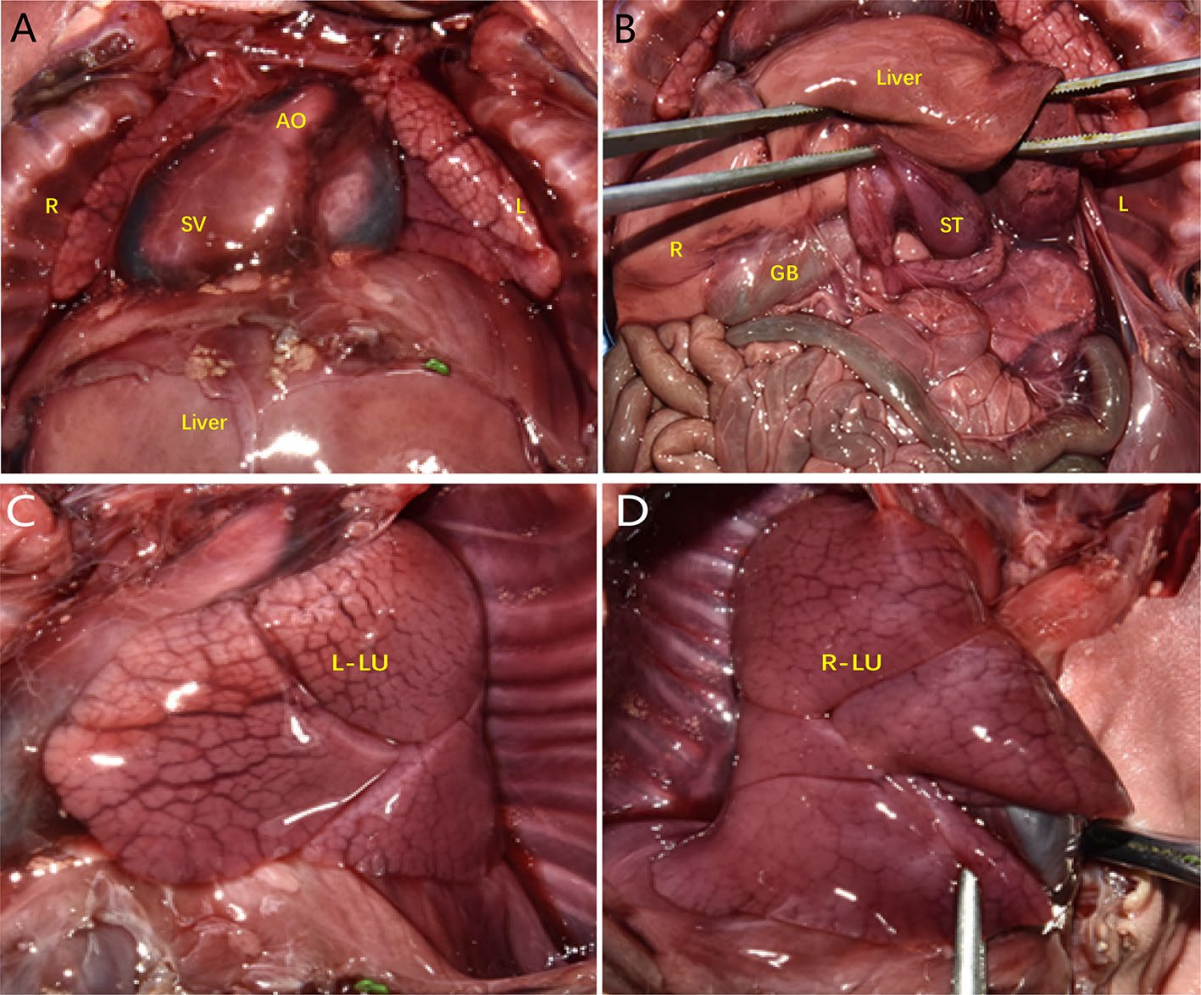
Examination of heart anatomy. (**A**, **B**) The apex of the heart was to the right, there was a single ventricle, the gallbladder and stomach were in the right and left abdomens, respectively, and a spleen was lacking. *GB* gallbladder, *L-LU* left lung, *R-LU* right lung, *ST* stomach.

**Figure 4:**
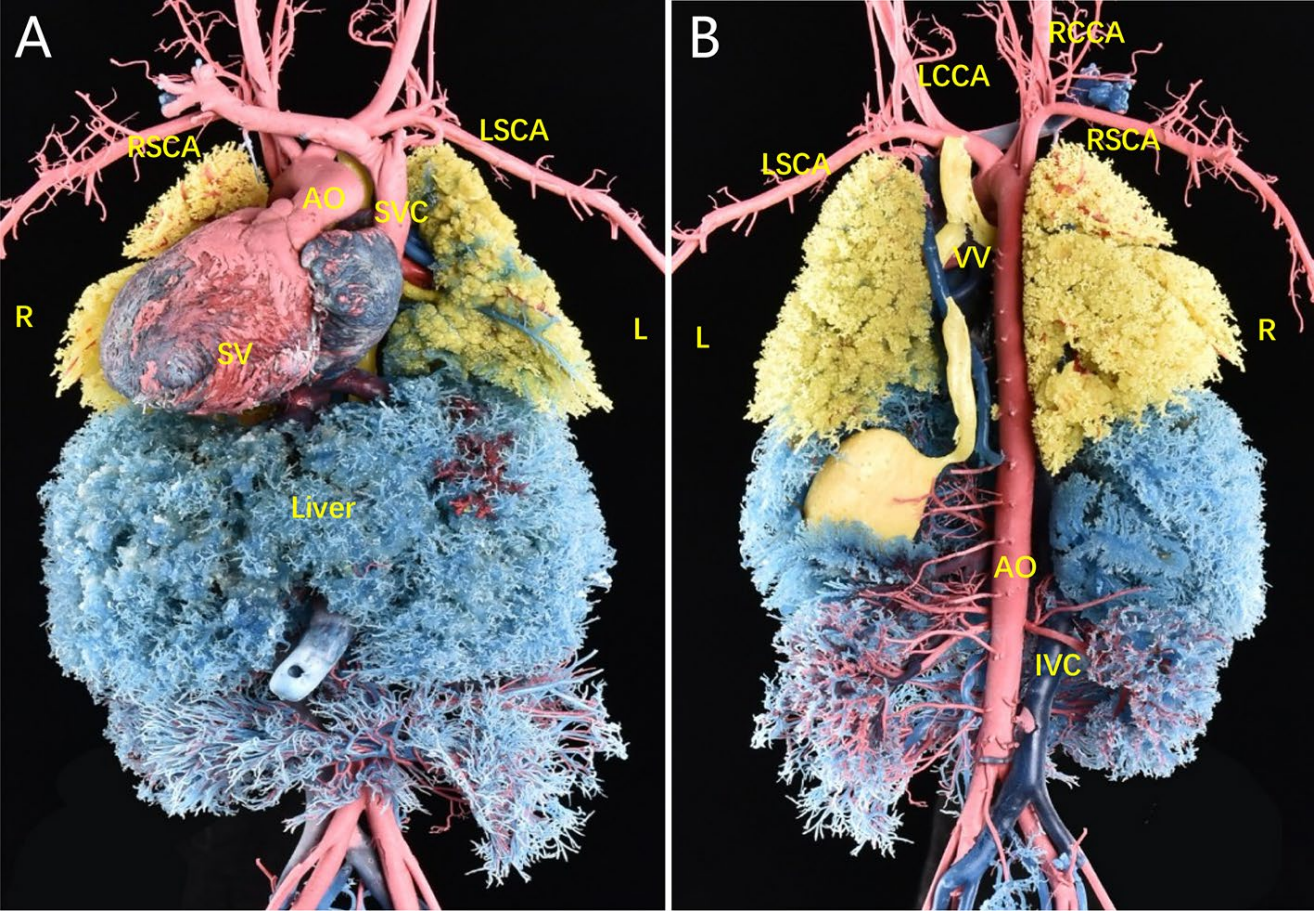
Confirmation of the prenatal diagnosis based on the cardiovascular cast. (**A**, **B**) The cast revealed dextrocardia, a single ventricle, isolated left superior vena cava, right aortic arch with mirror branches, and anomalous complete drainage of the PV into the left superior vena cava.

**Figure 5:**
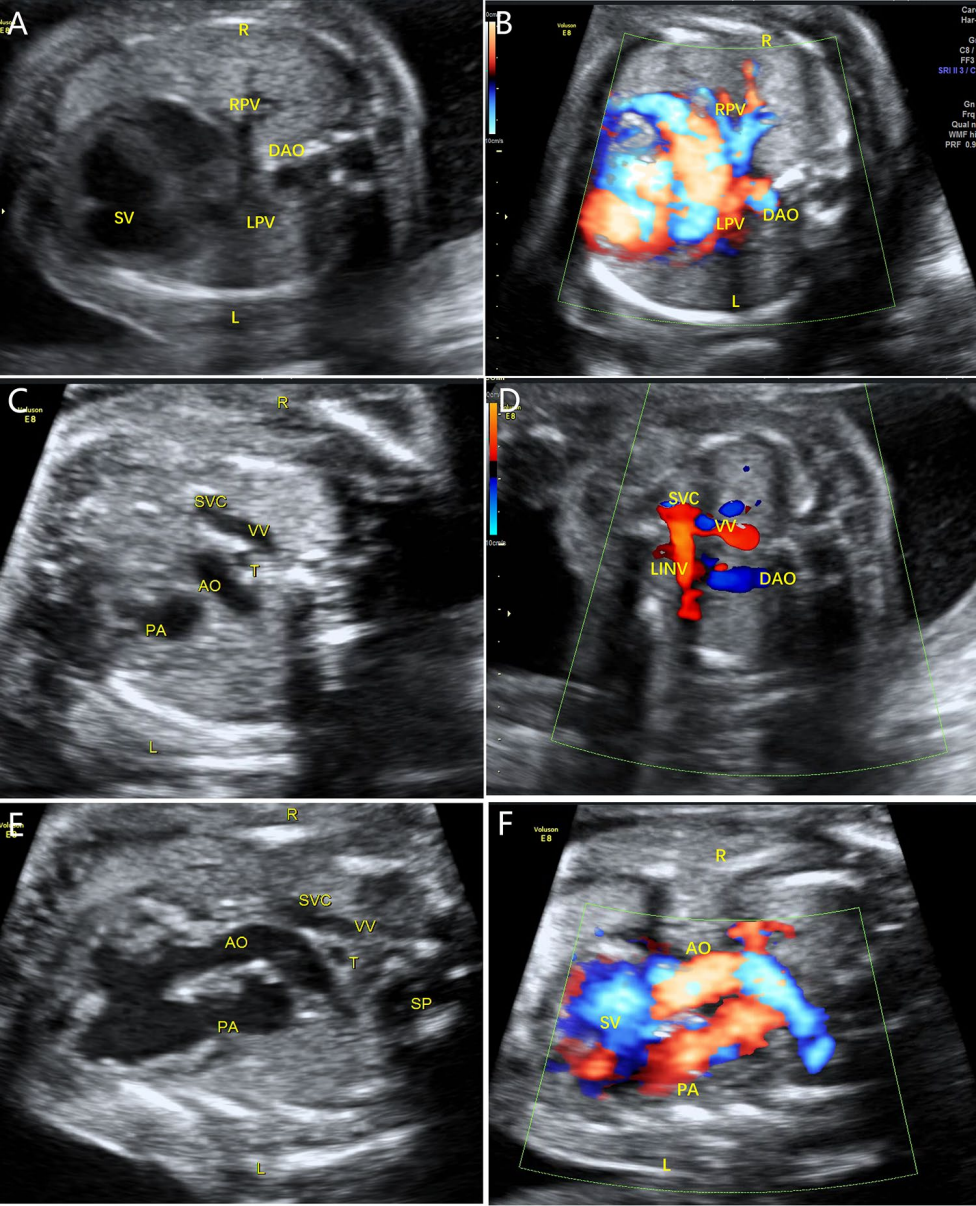
Prenatal ultrasound diagnosis of fetal supracardiac TAPVC with complex malformations. (**A**, **B**) Two-dimensional and color Doppler flow imaging showed the left and right PVs converging to form a common PV trunk, with an increased posterior left atrium index. (**C**, **D**) The common PV trunk returned to the right superior vena cava via the vertical vein in the view showing 3 vessels and trachea. (**E**, **F**) Aorta and pulmonary artery originating from a single ventricle. *LINV* left innominate vein, *SP* spine, *SVC* superior vena cava, *T* trachea, *VV* vertical vein.

**Figure 6:**
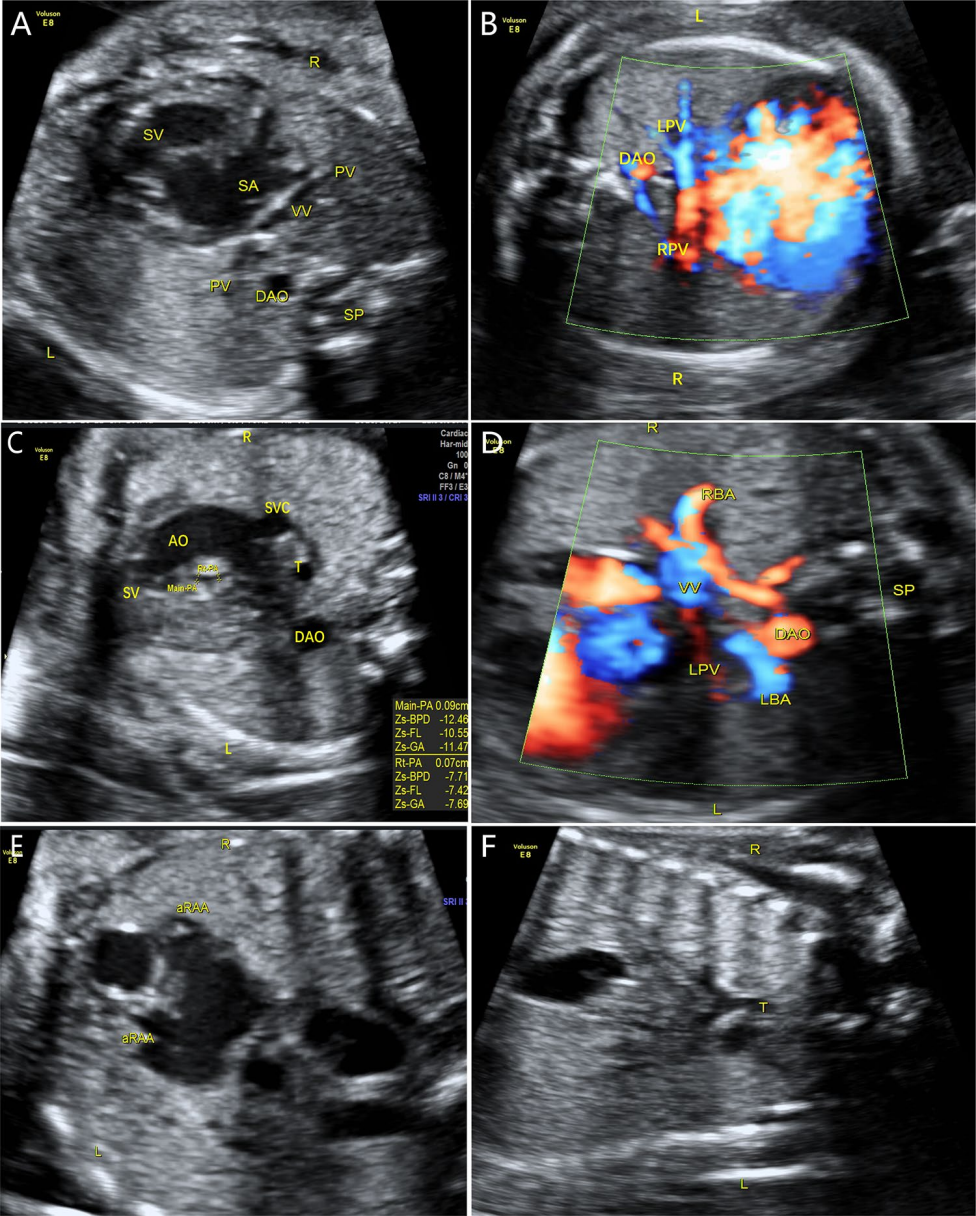
Prenatal ultrasound diagnosis of fetal infracardiac TAPVC with complex malformations. (**A**, **B**) Two-dimensional and color Doppler flow imaging showing a single atrium, single atrioventricular valve, single ventricle, and left and right PVs forming a common PV trunk, accompanied by increased posterior left atrium index. The pulmonary artery and aorta both originated from a single ventricle and pulmonary atresia was observed. (**C**, **D**) A collateral blood supply from the descending aorta in both lungs was seen in the cross section of the heart. (**E**) Both left and right atrial appendages were anatomically right atrial appendages. (**F**) Left and right main bronchi were symmetrical.

**Figure 7:**
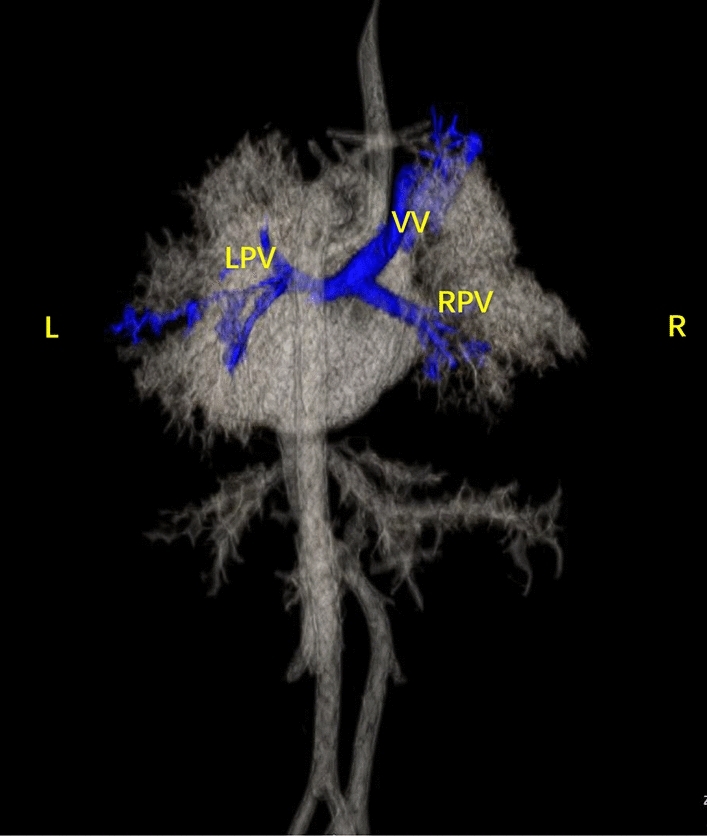
Postpartum CTA findings. The left and right PVs converged to form a PV trunk and returned to the right superior vena cava via the vertical vein.

**Figure 8:**
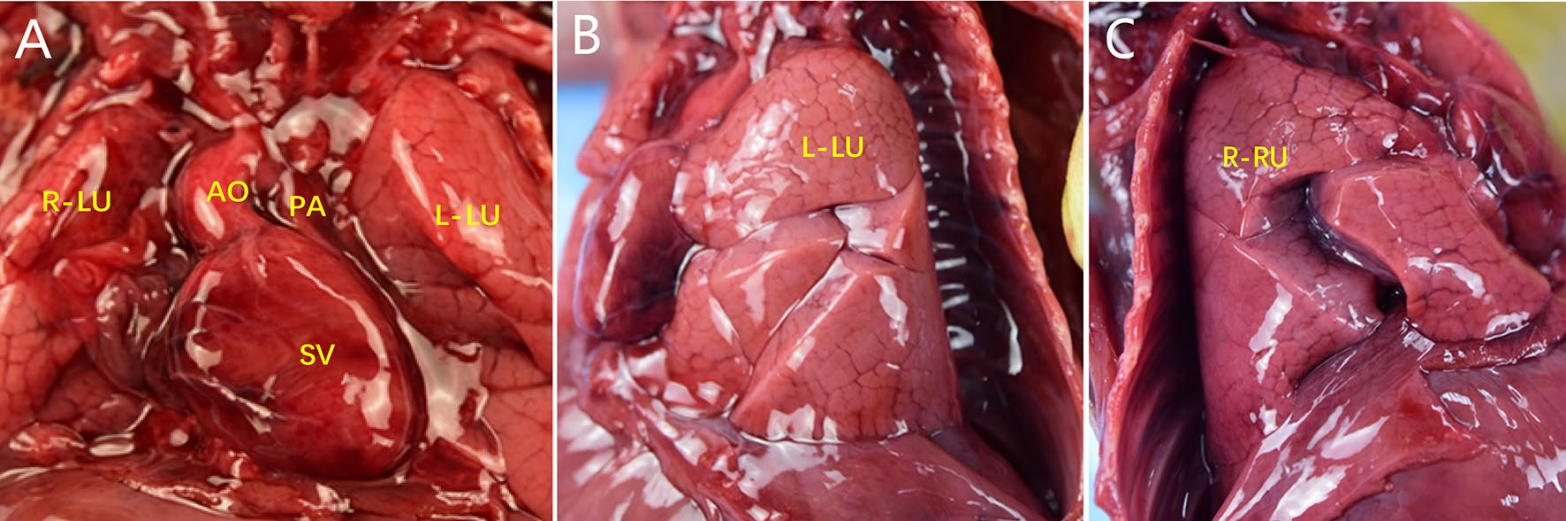
Examination of heart anatomy. (**A**–**C**) A single ventricle and bilateral trefoil-shaped lungs were observed.

**Figure 9:**
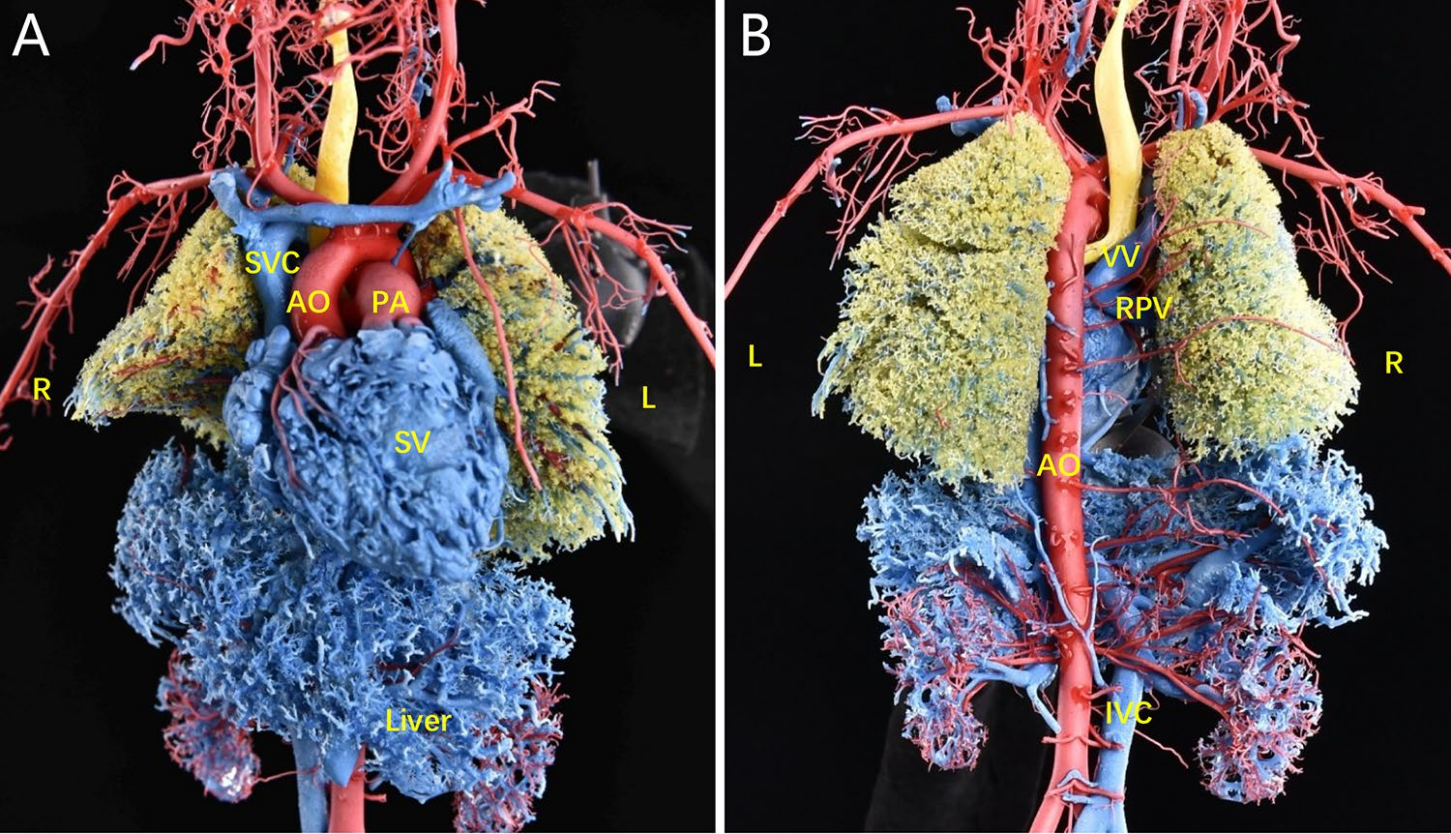
Findings from the postnatal cardiovascular cast. (**A**, **B**) A single ventricle with double outlet, and left aortic arch were observed, with the common PV trunk returning to the right superior vena cava via the vertical vein.

**Figure 10:**
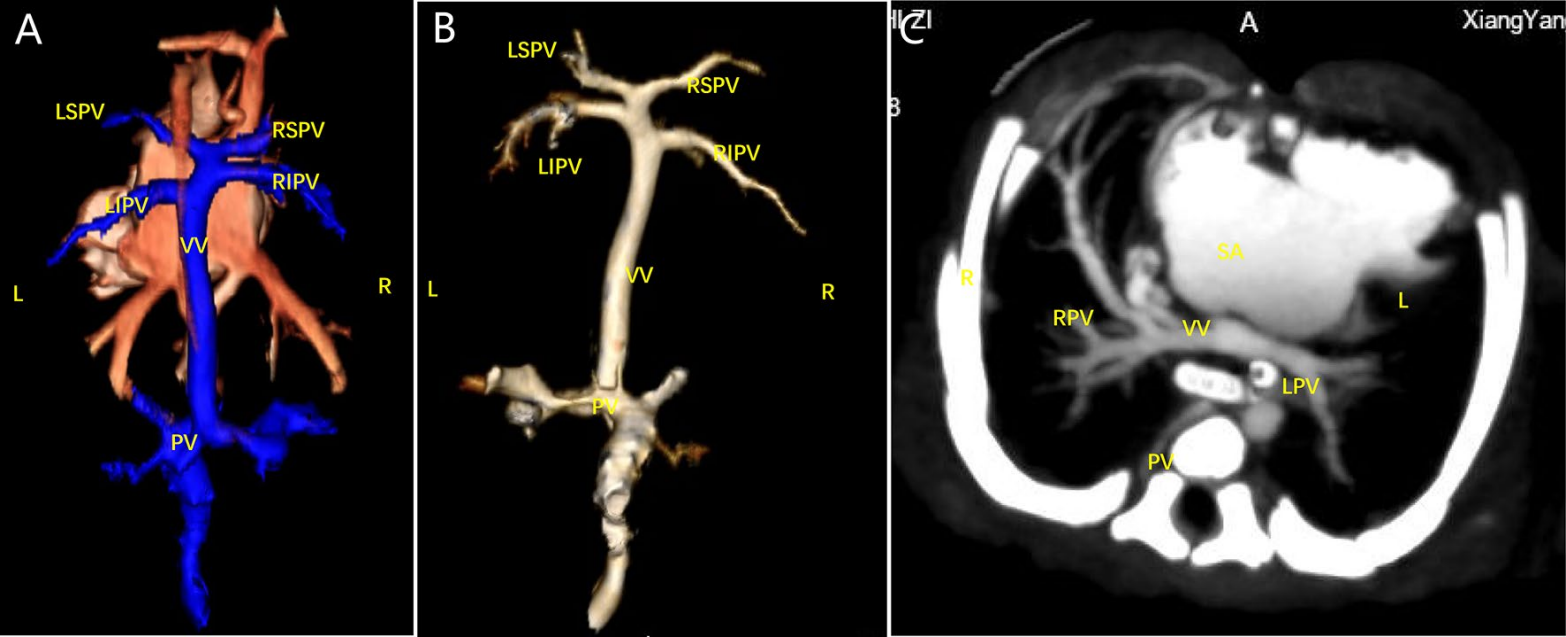
Postpartum CTA findings. (**A**–**C**) Left and right PVs converged to form a common PV trunk that flowed back to the portal vein via the vertical vein.

**Figure 11:**
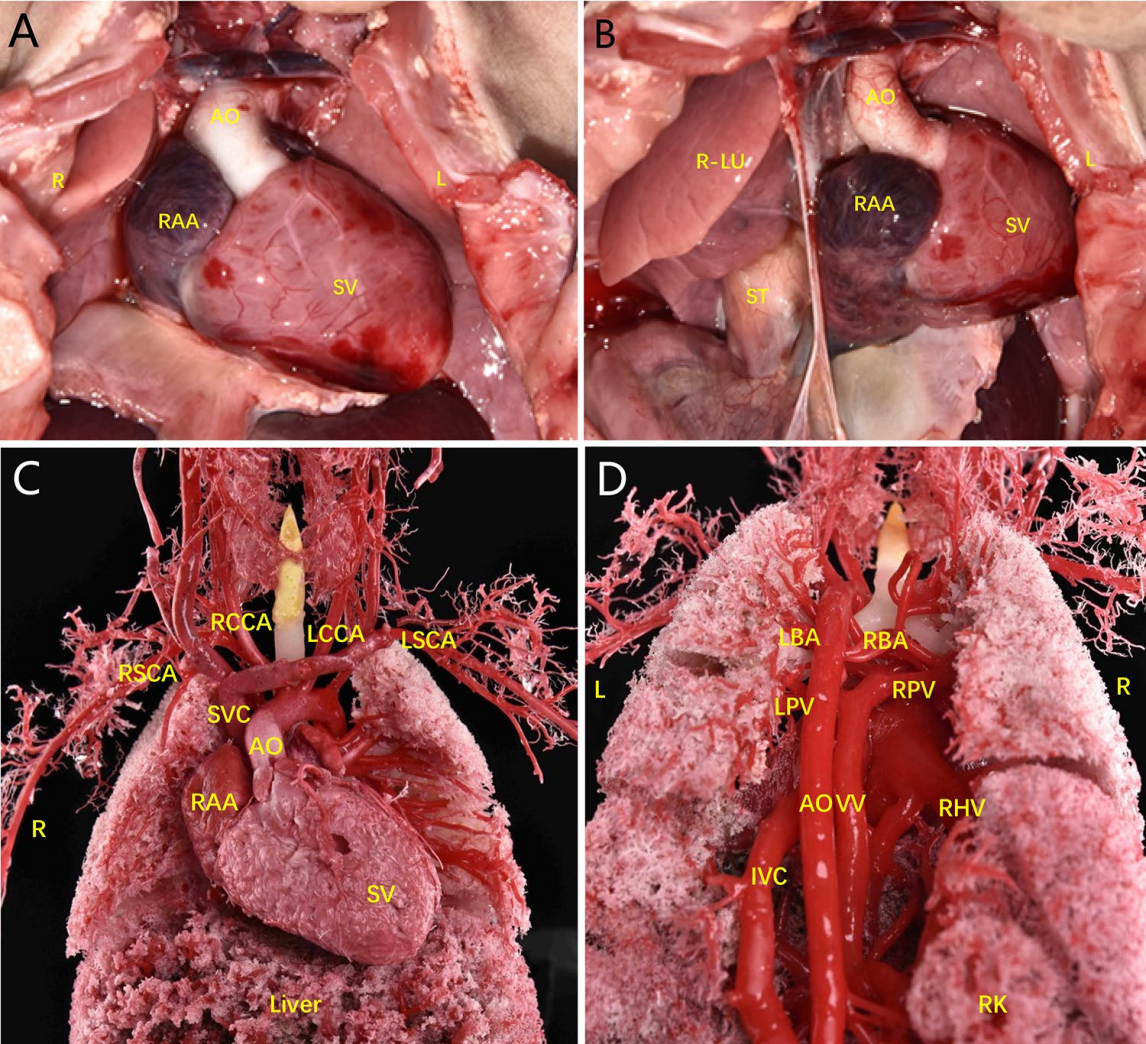
Postnatal anatomic and cardiovascular cast findings. (**A**, **B**) The prenatal examination revealed a single ventricle, pulmonary atresia, left and right collateral arteries from the descending aorta supplying both lungs, left and right PVs converging to form a PV trunk and descending into the portal vein via the vertical vein.

The common characteristics of fetal TAPVC in this study were a smaller left atrium in the 4-chamber view, no PV opening in the posterior wall of the left atrium, and extra vessels behind the left atrium in the 3-vessel trachea view. Modified cardiovascular casts were used in the postpartum diagnosis of 3 TAPVC cases, including 1 case of supracardiac and 2 of infracardiac TAPVC. For cardiac TAPVC, right atrial isomerism, a single atrium and ventricle, double-outlet single ventricle, pulmonary artery stenosis, right aortic arch with mirror branches, right descending aorta, slim ductus arteriosus, and dextrocardia were prenatally diagnosed (case 1, Fig. 1), which was confirmed by postpartum findings (Figs. 2, 3, 4). In the 2 cases of cardiac TAPVC, right atrial isomerism, diaphragmatic hernia, a single atrium, pulmonary atresia, abnormal inflow and atrial isomerism syndrome, levoversion, atrial septal defect, functional single ventricle, double-outlet single ventricle, and double-inlet single ventricle were prenatally diagnosed (case 8, Fig. 5; case 17, Fig. 6), which was confirmed postpartum (Figs. 7, 8, 9, 10, 11).

### PV drainage routes

Specific PV drainage routes and modified corrosion technique to the currently available fetal imaging methods on TAPVC were observed in the 18 cases of fetal TAPVC (Tables 2, 3). In the 8 cases of supracardiac TAPVC there was PV entry into the right superior vena cava via the vertical vein (n = 3), the left superior vena cava via the vertical vein (n = 3), and the right superior vena cava via the vertical and left brachiocephalic veins (n = 3). In the 4 infracardiac cases, PVs entered the portal vein and inferior vena cava via the vertical vein. In the 6 cases of the cardiac type, PVs entered the right atrium either via the coronary sinus (n = 2) or directly (n = 4). PV drainage in mixed TAPVC is more complex than in the other types and often occurs by 2 routes. This was observed in the present study. One of the routes involved entry of the left superior PV into the right superior vena cava via the vertical and left brachiocephalic veins, while the left inferior and right superior veins entered the right atrium via the coronary sinus. In the other route, the left inferior vein directly entered the left atrium while the left superior and right PVs entered the left superior vena cava via the vertical vein.

**Table 2:** Specific pulmonary vein drainage routes of 18 cases with confirmed fetal TAPVC.

Type of TAPVC	Drainage route of PV	Number
Supracardiac type	PV → VV → RSVC	3
	PV → VV → LSVC	3
	PV → VV → LBV → RSVC	2
Infracardiac type	PV → VV → portal vein	2
	PV → VV → IVC	2
Cardiac type	PV → RA	4
	PV → CS → RA	2
Mixed type	LSPV → VV → LBV → RSVC/LIPV,RSPV → CS → RA	1
	LIPV → LA/LSPV,RPV → VV → LSVC	1

*PV* pulmonary vein, *VV* vertical vein, *RSVC* right superior vena cava, *LSVC* left superior vena cava, *LBV* Left brachiocephalic vein, *IVC* Inferior vena cava, *RA* right atrium, *CS* coronary sinus, *LSPV* left superior pulmonary vein, *LIPV* left inferior pulmonary vein, *LA* left atrium, *RPV* right pulmonary vein.

**Table 3:** Comparing the modified corrosion technique to the currently available fetal imaging methods on TAPVC.

Methods	Characteristics of vascular display	Cases of pulmonary veins better delineated
Modified corrosion technique	Cardiovascular casting technique has its own superiority in exhibiting TAPVC abnormalities, especially in certain types such as course, origin and absence abnormalities of ductus	All cases of pulmonary veins are perfectly delineated
Autopsy	For complex congenital heart malformations, the display of vessels course is not as good as that of cardiovascular casting	8
Prenatal ultrasound	Prenatal ultrasound is limited by maternal amniotic fluid content, fetal position and ultrasound imaging itself, which is easy to cause missed diagnosis and misdiagnosis of TAPVC	The diagnosis of 1 case each of supracardiac and cardiac TAPVC was modified to partial anomalous pulmonary venous connection; additionally, 4 malformations were missed and 2 were misdiagnosed
Postnatal echocardiography	It can't display the complex vascular malformation stereoscopically and completely	12
Postpartum computed tomography angiography	The imaging results is closely related to the effect of cadaveric radiography after induced labor, which is prone to the reconstruction artifacts caused by the overflow of contrast medium	5

## Discussion

TAPVC can be accurately diagnosed after birth by combining 2-dimensional echocardiography with color Doppler imaging. However, prenatal diagnosis remains a clinical challenge, and there have few studies addressing this issue^11,15,16^. In the early stage of pregnancy, fetal blood mostly circulates through the ductus arteriosus and only a small portion of pulmonary blood flows into the left atrium via the PV; as no hemodynamic changes are observed at this time, diagnosing TAPVC is difficult. During the middle and late periods of pregnancy, pulmonary blood flow into the right atrium via the PV is increased, including in the fetus with TAPVC. At this point, pulmonary hypertension can easily occur and can lead to right heart failure postnatally and death. Therefore, prenatal diagnosis of fetal TAPVC is extremely important^13,17,18^.

The results of this retrospective study carried out at our Maternal–Fetal Medical Center showed that TAPVC can be identified by targeted fetal echocardiography, which can provide important 2D and spectral information that can be used for diagnosis. Moreover, we demonstrated that a modified cardiovascular cast can be used to display the 3D structure of the PV, which can also guide prenatal diagnosis. Among the 20 fetuses with a prenatal diagnosis of TAPVC, 18 were confirmed by postpartum diagnosis, and 2 cases were modified to PAPVC. Both of these showed complex intracardiac anatomy, with right atrial isomerism, endocardial cushion defect, a single ventricle, interrupted aortic arch, and PAPVC with the right PV connected to the right superior vena cava observed in 1 case and right atrial isomerism, endocardial cushion defect, functional single ventricle, double-outlet single ventricle, pulmonary artery stenosis, double superior vena cava, and PAPVC with the right PV connected to the right atrium observed in the other. Besides the 4 cases of isolated TAPVC, 14/18 cases (78%) were complicated by intra- or extracardiac malformations, and 61% (11/18) were complicated by heterotaxia syndrome; as the latter can lead to atrial heterogeneity and a change in the position of the atrium, it is critical for clinicians to identify the true left and right atria, which may be a key step in the diagnosis of TAPVC. Following pre- and postnatal diagnoses, fetal echocardiography showed no direct connection between the PV and left atrium but revealed the convergence of PVs at the back of the atrium to form a common vein stem; an increased distance between the posterior wall of the atrium and descending aorta; and a visible ascending or descending vertical vein. These features not only exist in a fetus with complex intra- or extracardiac malformations, but also in isolated TAPVC.

Our modified cardiovascular cast is another method for postmortem evaluation of fetal TAPVC. In China, our team was the first to improve and patent the modified vascular casting technology. We previously demonstrated that the cardiovascular cast can accurately show various complex fetal cardiac malformations, which can facilitate prenatal diagnosis and reduce the risk of overlooking or misdiagnosing the Complex cardiac malformations^19,20^.

Several studies have evaluated the clinical utility of postmortem magnetic resonance imaging (MRI) and micro-CT^21–23^.

However, casting may better reveal some rare malformations, especially smaller branching anomalies that are difficult to detect by other imaging modalities. In the future, this new approach of casting combined with micro-CT or MRI may be used to develop 3D digital models for clinical assessment and teaching.

## Conclusion

The results of our study demonstrate that prenatal ultrasound can be used to accurately diagnose most cases of fetal TAPVC. By analyzing the echocardiography findings, the specific type of fetal TAPVC can be identified. Identifying the PV drainage route is critical for diagnosing different types of TAPVC. Finally, a postpartum cardiovascular cast can accurately depict the branch structure of the heart’s major vessels, which can guide prenatal diagnosis of TAPVC for timely management that can lead to an improved outcome.

## Data Availability

The data used to support the findings of this study are included within the article.
